# CAFET Algorithm Reveals Wnt/PCP Signature in Lung Squamous Cell Carcinoma

**DOI:** 10.1371/journal.pone.0025807

**Published:** 2011-10-10

**Authors:** Yue Hu, Anna V. Galkin, Chunlei Wu, Venkateshwar Reddy, Andrew I. Su

**Affiliations:** Genomics Institute of the Novartis Research Foundation, San Diego, California, United States of America; Univesity of Texas Southwestern Medical Center at Dallas, United States of America

## Abstract

We analyzed the gene expression patterns of 138 Non-Small Cell Lung Cancer (**NSCLC**) samples and developed a new algorithm called Coverage Analysis with Fisher’s Exact Test (**CAFET**) to identify molecular pathways that are differentially activated in squamous cell carcinoma (**SCC**) and adenocarcinoma (**AC**) subtypes. Analysis of the lung cancer samples demonstrated hierarchical clustering according to the histological subtype and revealed a strong enrichment for the Wnt signaling pathway components in the cluster consisting predominantly of SCC samples. The specific gene expression pattern observed correlated with enhanced activation of the Wnt Planar Cell Polarity (**PCP**) pathway and inhibition of the canonical Wnt signaling branch. Further real time RT-PCR follow-up with additional primary tumor samples and lung cancer cell lines confirmed enrichment of Wnt/PCP pathway associated genes in the SCC subtype. Dysregulation of the canonical Wnt pathway, characterized by increased levels of β-catenin and epigenetic silencing of negative regulators, has been reported in adenocarcinoma of the lung. Our results suggest that SCC and AC utilize different branches of the Wnt pathway during oncogenesis.

## Introduction

Lung cancer is the leading cause of cancer-related death in both men and women throughout the world, and more than fifteen thousand people in the United States die from the disease each year [1]. About 80% of lung cancers are classified as non-small cell lung carcinoma (**NSCLC**). Adenocarcinoma (**AC**) and squamous cell carcinoma (**SCC**) are the two major subtypes of NSCLC, each representing about 40% cases of NSCLC. SCC is characterized as a poorly differentiated tumor subtype that develops in the proximal airways and is strongly associated with cigarette smoking. In contrast, AC usually arises in the peripheral airways and is more commonly observed in non-smokers and women.

High-throughput gene expression analysis has been widely used to study cancer to facilitate the discovery of novel oncogenes and elucidate the mechanism of tumorigenesis. These genome-wide analyses usually result in the identification of hundreds or thousands of genes with an altered expression pattern. However, interpreting the relevance of these long gene lists remains a significant challenge [2], [3].

Several pathway analysis approaches have been developed to uncover the molecular signaling patterns underlying these candidate gene lists. One of the most common approaches is based on statistical enrichment (e.g., hypergeometric distribution with the Fisher's Exact Test). These methods test the gene list of interest for enrichment relative to groups of genes that are known to share a common function. This approach, broadly referred to here as functional group enrichment analysis (**FGA**), calculates the statistical significance of the overlap with the goal of identifying activated or repressed pathways. This basic method is used in many major pathway analysis tools including Ingenuity, Database for Annotation, Visualization and Integrated Discovery (**DAVID**), and gene set enrichment analysis (**GSEA**) [4], [5]. These tools have been successfully applied to generate molecular insights in many biological systems.

In this study, we analyzed a collection of 138 lung cancer samples using an FGA approach with the goal of defining the active pathways that differentiate the two major sample groups. While developmental and cell cycle pathways were broadly implicated, this approach was unable to identify specific molecular pathways that were amenable to hypothesis testing. In an effort to identify more precise pathways that were dysregulated in this data set, we developed a new algorithm called Coverage Analysis with Fisher’s Exact Test (**CAFET**). This algorithm specifically accounts for the case where dysregulation of even a single pathway member can result in altered pathway signaling. Using the CAFET approach, we found that Wnt pathway components were differentially expressed in SCC samples. Further characterization of these samples revealed an inhibition of the canonical branch of the Wnt pathway, coupled with an enhancement of the non-canonical Wnt PCP signaling cascade. These results suggest that lung SCC uses an alternate branch of the Wnt pathway for survival and development.

## Materials and Methods

### Gene expression data and analysis

Microarray gene expression data from 62 human lung AC and 76 lung SCC were downloaded from NCBI's GEO (GSE8894). Probe sets with a maximum intensity below 100 were removed. Hierarchical clustering was performed with R using a Euclidean distance metric and average linkage. The significance of differential expression for each gene was evaluated using the two primary clusters from the global clustering analysis. The false discovery rate (FDR) was estimated using the Benjamini Hochberg method [6]. Genes were defined as differentially expressed if at least one probe had a FDR<0.05 and a mean difference greater than 2.5-fold between the two groups (**Tables S1** and **S2**).

Microarray data from a second lung cancer expression study (GSE10245) comprised of 58 NSCLC samples (40 AC and 18 SCC) were also analyzed and processed in the same way as above.

### Functional group enrichment analysis (FGA)

Functional gene sets were downloaded from two sources. Human gene annotations were obtained from NCBI's gene2go table (June19, 2009 snapshot from ftp://ftp.ncbi.nih.gov/gene/DATA/gene2go.gz), from which 10102 gene sets were extracted with at least five genes above the maximum intensity threshold in our data set. We also utilized the KEGG metabolic and signaling pathways database, which contained 202 manually-annotated human pathways with the same gene expression threshold (June 19, 2009 snapshot from ftp://ftp.genome.jp/pub/kegg/pathways).

In this study, we calculated FGA enrichment using Fisher’s exact test and hypergeometric distribution. The p-value for the enrichment of a gene set of N_G_ genes and a functional group of N_F_ genes was calculated by:
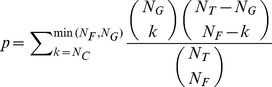
(1)


**Figure 1:**
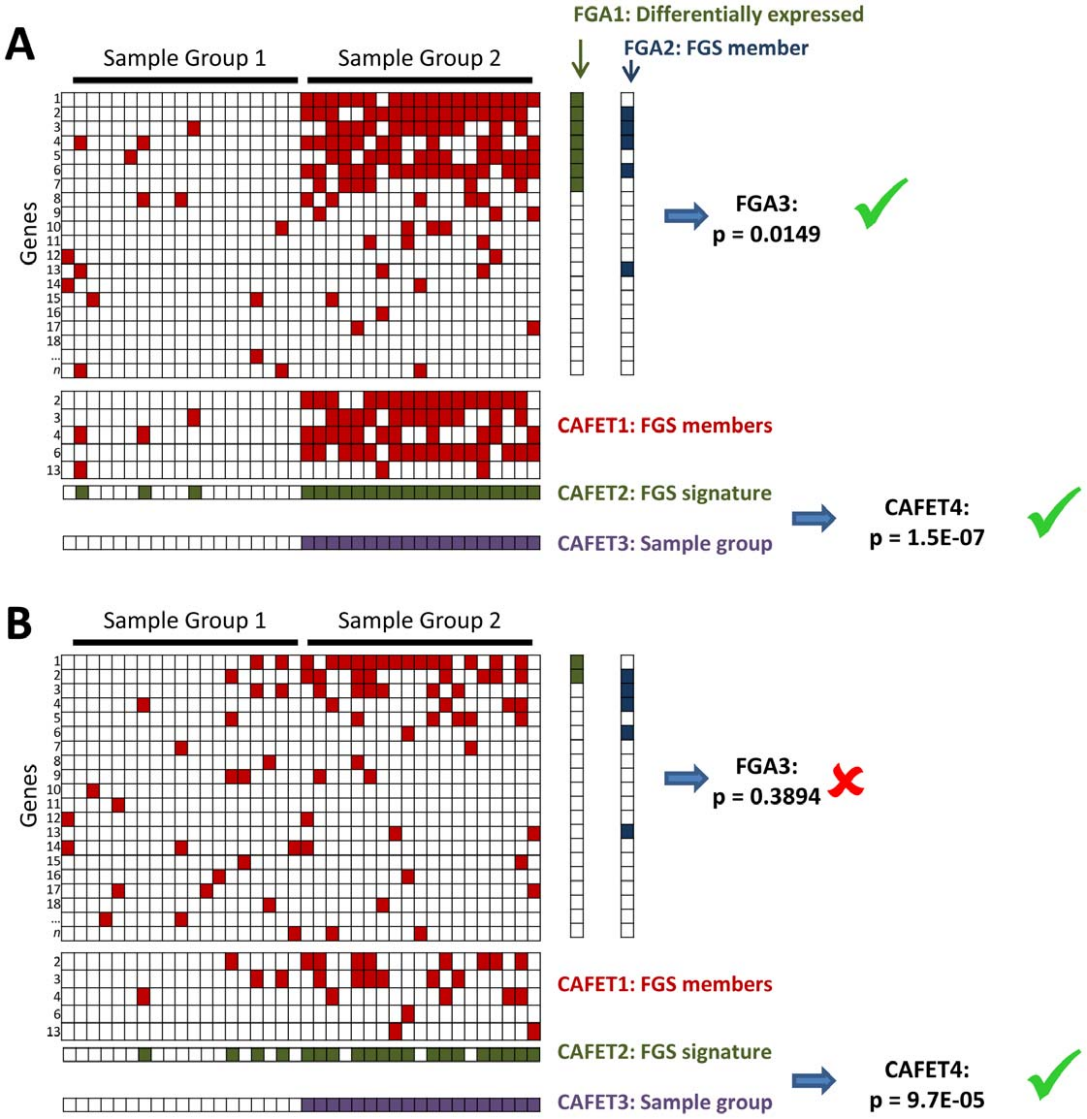
Schematic illustration of the FGA and CAFET approaches for pathway enrichment. Both the FGA and CAFET approaches begin with the same data matrix of gene expression measurements, and both seek to assess the relevance of a particular Functional Gene Set (FGS) (i.e., pathway) in the division of samples into two groups. Red boxes indicate dysregulation of a specific gene in a specific sample. FGA approaches employ a three-step process. In step 1, differentially expressed genes are identified, typically based on a t-test or ANOVA analysis. In step 2, genes with a role the FGS of interest are identified. In step 3, Fisher’s exact test is used to test for enrichment of FGS genes among differentially-expressed genes. CAFET employs a similar four-step process. In step 1, FGS genes are first identified and the corresponding sub-matrix is extracted. In step 2, samples are evaluated for the presence of a particular gene expression signature. In this study, the signature is marked as present if one or more pathway genes are dysregulated. In step 3, the division of samples between two groups of interest is defined. In step 4, Fisher’s exact test is used to test for enrichment of samples containing the pathway signature among the sample group of interest. In cases where the majority of FGS members are differentially regulated (**A**), both FGA and CAFET detect a statistically significant relationship between the FGS and the sample grouping. However, in cases where each sample has only a few FGS members dysregulated (**B**), CAFET but not FGA results in a significant enrichment for the associated FGS.

Where N_C_ is defined as the number of genes within the gene set assigned to the functional group, N_T_ is the total number of annotated genes of microarray. The FGA approach is also illustrated schematically in **Figure 1**. FDR was estimated using the Benjamini-Hochberg method, and a threshold of 0.05 was applied.

### Coverage Analysis with Fisher’s Exact Test (CAFET)

CAFET calculates the degree to which samples with a desired expression property were concentrated in a particular sample cluster. In this study, the desired property for a single gene in a single sample was its overexpression relative to the median expression across all samples. Like the FGA, the CAFET metric is based on the Fisher’s exact test and hypergeometric distribution. However, instead of calculating the enrichment of genes as in FGA, CAFET calculates the enrichment of samples with a certain expression feature.

The p-value of CAFET for a single gene is calculated by:
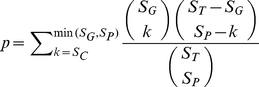
(2)where S_G_ is the number of samples in the sample cluster of interest, S_T_ is the total number of samples, S_P_ is the number of samples with the expression pattern of interest, and S_C_ is the number of samples in S_P_ that fall in S_G_. Our expression filtering criterion focused on expression greater than 2.5-fold of the median expression across of all samples, or on expression less than 0.5 fold of the median. (Asymmetric thresholds were used because the baseline noise limits the magnitude of down-regulation.) For any gene with multiple probes, all those samples with at least one probe reaching the criteria were taken into account. FDR was again estimated using the Benjamini-Hochberg method, and a threshold of 0.05 was applied.

A CAFET p-value and FDR can be calculated for a single gene (as described above) or for an entire functional group. In the latter case, the desired property is overexpression of any gene in the functional group (illustrated schematically in **Figure 1**). For each gene *i* = 1 … *n* in a given functional group, S_P(i)_ and S_C(i)_ were defined as the S_C_ and S_P_ in formula 2. We further defined S_FP_ as the union of samples sets for S_P(i)_ for all gene *i*, *i* = 1 … *n*, and S_FC_ as the number of union of samples sets for S_C(i)_ for all gene *i*, 1 … *n*. The CAFET p-value of the functional group was then calculated by:
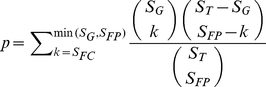
(3)


FDR was then estimated as described previously. Only functional groups with at least 5 genes with 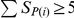 were considered.

### Score of Wnt SCC signature

To assess the degree to which individual samples exhibited dysregulation of the Wnt pathway, we developed an ad hoc scoring function based on gene expression values. A normalized expression value is calculated for each gene based on the average intensity of all its probes after log2 transformation and standardization. The score of the Wnt SCC signature for any sample is the sum of expression values of upregulated genes subtracting the sum of expression values of down-regulated genes.

### Primary Tumor and Cancer Cell Line Samples

cDNA from 40 lung cancer samples (14 SCC and 26 non-SCC) was obtained from Origene (Rockville, MD; product #HLRT103). Expression of *FZD6*, *DVL3*, and *WNT5A* was interrogated via RT-PCR according to the protocol below.

Total RNA from 12 primary human lung tumors and 12 matched normal tissue samples were obtained from Asterand (Detroit, MI). Informed consent was obtained from patients by Asterand under approval from the appropriate IRBs. The samples were handled and maintained according to protocols approved by the IRB of the Genomics Institute of the Novartis Research Foundation (GNF).

SCC lung cancer cell lines LK2 (RIKEN, Japan), NCI-H520 (ATCC, Monassas, VA), LUDLU-1 (ECACC, UK) and HARA-1 (HSSRB, Japan) were maintained in HyClone RPMI-1640 medium supplemented with 10% FBS (Thermo Fisher Scientific Inc., Waltham, MA). Non-SCC lung cancer cell lines ABC1 (HSSRB, Japan), PC14 (RIKEN, Japan), NCI-H2342, NCI-H209, A549, NCI-H661, HCC827 and NCI-H522 (ATCC, Monassas, VA) were maintained in the recommended media by their respective cell banks. Trizol reagent (Invitrogen, Carlsbad, CA) was used to extract total RNA from cancer cell lines.

### Real-Time PCR Assays

For expression analysis, cDNA was prepared using the High Capacity cDNA Archive Kit (Applied Biosystems, Foster City, CA). All RT-PCR assays were performed in duplicate using pre-designed gene-specific Taqman probes and Taqman Universal PCR Master Mix (Applied Biosystems, Foster City, CA) on the 7900HT FAST Real-Time PCR System. Relative mRNA expression of target genes was normalized to *ACTB* expression as an internal amplification control.

## Results

**Figure 2:**
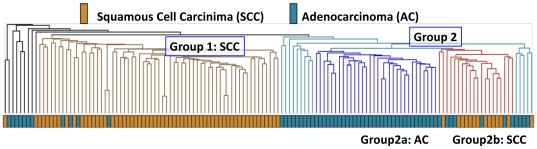
Hierarchical clustering of 138 NSCLC samples reveals two predominant sample clusters. Hierarchical clustering was performed based on log-transformed expression data using Euclidean distance and average linkage. The brown branches of the tree were labeled “Group 1”, and the light blue cluster was labeled “Group 2”. Group 2 was further subdivided into Group 2a (dark blue) and Group 2b (red).

We analyzed gene expression data from 138 NSCLC samples, classified into 76 SCC and 62 AC tumors [7]. Hierarchical clustering separated the majority of the samples into two branches, which we labeled as simply Group 1 and Group 2. Group 1was primarily comprised of SCC samples (59 SCC, 4AC). Group 2 was further subdivided into Group 2a, which contained only AC samples, and Group 2b, which contained the majority of the remaining SCC samples that were not found in Group 1 (**Figure 2**). Recognizing that lung cancer is a very heterogeneous disease even within histological classes, we specifically chose to use the global, unsupervised clustering results as the basis for our study. Specifically, we focused on identifying the molecular basis distinguishing Group 1 from Group 2 lung cancer samples.

We identified 635 genes with significantly higher expression in Group 1 (**Table S1**), and 740 genes with significantly higher expression in Group 2 (**Table S2**). To identify relevant pathways in these gene lists, we applied FGA analysis to these sets of genes. We found that genes overexpressed in Group 1 were enriched in functional groups related to cell cycle and development (**Table S3**), while genes overexpressed in Group 2 had significant association with many immune response functional groups (**Table S4**).

Enrichment in functional groups related to cell cycle and development was expected given their known roles in oncogenesis and metastasis. However, the functional gene groups identified in this analysis often contained hundreds or thousands of genes, and as a result, the formulation of specific mechanistic hypotheses proved difficult. Enrichment scores of more specific signaling pathways using FGA were not statistically significant, and varying filtering criteria for differential expression did not result in any improvement.

We hypothesized that this lack of specificity was a fundamental property of the enrichment analysis underlying FGA. Specifically, the FGA approach is designed to detect pathways in which multiple pathway genes are differentially expressed, and more significant p-values are achieved when more pathway genes are differentially expressed (**Figure 1**). The first step in FGA is the identification of differentially expressed genes, typically involving a statistical measure like a t-test or ANOVA. The second step involves identifying the set of pathway genes for a pathway of interest, and third step tests the enrichment of pathway genes among differentially expressed genes. This procedure is quite effective when the majority of pathway genes are differentially expressed in each case sample studied (**Figure 1A**).

However, dysregulation of even a single pathway gene is often sufficient to result in altered pathway signaling. Consider a study in which all case samples have altered pathway activity, but where each sample has a different pathway member dysregulated (**Figure 1B**). In this case, FGA will not detect the importance of the altered pathway.

To address this limitation, we developed a complementary algorithm called Coverage Analysis with Fisher’s Exact Test (**CAFET**). In the first step, data for pathway members are extracted from the gene expression matrix. In the second key step, CAFET identifies samples with a relevant gene expression signature, which in our case can be defined by the dysregulation of as few as one pathway member. The third step defines the clinically relevant sample groups (e.g., case versus control, or SCC versus AC). And the fourth step tests the enrichment of dysregulated samples among the sample groups using the same Fisher’s exact test as in FGA (**Figure 1**). The CAFET procedure is sensitive to data sets in which the majority of pathway members are dysregulated (**Figure 1A**), as well less obvious cases in which only one or a few pathway members are dysregulated (**Figure 1B**). In contrast to FGA where more significant p-values are achieved when more pathway genes are differentially expressed, CAFET reports more significant p-values when more samples have at least one differentially expressed pathway member.

We used CAFET to identify functional groups which significantly differentiated Group 1 from Group 2. Consistent with the FGA analysis, Group 1 samples showed significant CAFET enrichment for many developmental process related functional groups (**Table 1** **;** full results in **Tables S5** and **S6**). As designed, this list also included specific enriched molecular pathways. The Wnt receptor activity pathway (GO:0042813) was among the most enriched functional groups (p = 8.55E−13, FDR = 7.94E−11), with ninety lung cancer samples expressing at least one of the following seven components (*FZD3*; *FZD4*; *FZD6*; *FZD7*; *FZD8*; *FZD10*; *RYK*) at least 2.5-fold above the average of Group 2 samples. Sixty of these ninety samples were found to cluster with Group 1, indicating a strong association of the Wnt pathway. Given the known roles for Wnt signaling in oncogenesis and metastasis, we focused our study on the Wnt pathway to further investigate its potential dysregulation in the two main NSCLC subtypes [8], [9].

**Table 1:** Most significant functional groups identified by CAFET based on genes overexpressed in Group 1 samples.

FG_ID	FG_Name	Gene No.^a^	Total No.(138)^b^	G1 No. (63)^c^	G1_pValue	G1 FDR
GO:0030299	intestinal cholesterol absorption	5	79	60	2.08E−18	1.02E−15
hsa03030	DNA replication - Homo sapiens (human)	33	87	62	1.58E−17	5.08E−15
GO:0001840	neural plate development	5	70	55	3.93E−16	9.87E−14
GO:0000777	condensed chromosome kinetochore	40	92	62	4.92E−15	8.75E−13
GO:0005663	DNA replication factor C complex	6	57	48	5.43E−15	9.48E−13
GO:0048854	brain morphogenesis	7	77	57	6.03E−15	1.02E−12
GO:0033170	protein-DNA loading ATPase activity	5	56	47	2.35E−14	3.37E−12
GO:0003689	DNA clamp loader activity	5	56	47	2.35E−14	3.37E−12
GO:0006297	nucleotide-excision repair, DNA gap filling	17	81	58	2.95E−14	4.10E−12
GO:0048048	embryonic eye morphogenesis	7	76	56	3.98E−14	5.47E−12
GO:0035058	sensory cilium assembly	5	59	48	1.13E−13	1.39E−11
GO:0000779	condensed chromosome, centromeric region	43	95	62	1.17E−13	1.40E−11
GO:0007618	mating	5	80	57	2.06E−13	2.44E−11
GO:0051588	regulation of neurotransmitter transport	5	92	61	2.24E−13	2.60E−11
GO:0046928	regulation of neurotransmitter secretion	5	92	61	2.24E−13	2.60E−11
GO:0030057	desmosome	19	101	63	6.46E−13	6.17E−11
hsa03430	Mismatch repair - Homo sapiens (human)	21	84	58	8.22E−13	7.77E−11
GO:0006596	polyamine biosynthetic process	5	49	42	8.37E−13	7.84E−11
GO:0042813	Wnt receptor activity	7	90	60	8.55E−13	7.94E−11
GO:0005657	replication fork	28	97	62	8.74E−13	7.97E−11
GO:0002347	response to tumor cell	5	97	62	8.74E−13	7.97E−11
GO:0070567	cytidylyltransferase activity	6	52	43	5.09E−12	3.98E−10
GO:0005871	kinesin complex	12	73	53	5.85E−12	4.47E−10
GO:0031507	heterochromatin formation	5	73	53	5.85E−12	4.47E−10
GO:0008088	axon cargo transport	10	92	60	6.36E−12	4.78E−10
GO:0042490	mechanoreceptor differentiation	9	92	60	6.36E−12	4.78E−10
GO:0042491	auditory receptor cell differentiation	6	71	52	8.50E−12	6.30E−10
GO:0050892	intestinal absorption	8	96	61	1.19E−11	8.76E−10

a : number of genes in the functional group with at least one overexpressed sample.

b : the total number of samples overexpressing at least one gene in the functional group (out of 138 samples total).

c : the number of Group 1 samples overexpressing at least one gene in the functional group (out of 63 samples in Group 1).

We first expanded our list of Wnt pathway members to 172 genes in nine Wnt-related functional groups (**Tables S7** and **S8**), and then applied CAFET on a gene-by-gene basis, testing whether samples with substantially altered expression were enriched among the SCC-dominated Group 1. This approach identified 53 genes that displayed differential expression and could be associated specifically with the Group 1 cluster (**Table 2**). Of these 53 genes, 34 were observed to be strongly up-regulated in Group 1, while 19 displayed significantly reduced expression levels. According to the CAFET method, *SOX2* was the most significantly enriched gene in the SCC cluster (FDR = 3.86E−18), with eighty eight percent of samples with high *SOX2* expression (54 of 61) clustering to Group 1. *SOX2* has recently been identified as a lineage survival oncogene in lung and esophageal squamous carcinoma [10]–[12].

**Table 2:** Genes in the Wnt pathway with significant CAFET enrichment.

Up-regulation				Down-regulation			
Gene	Total No^a^	G1 No^b^	G1_FDR	Gene	Total No^c^	G1 No^d^	G1_FDR
SOX2	61	54	3.86E−18	TLE2	39	33	2.94E−07
FZD10	60	53	1.67E−17	FZD5	35	28	6.08E−05
VANGL2	42	41	1.98E−15	CTBP1	21	19	0.000138
FZD7	46	42	4.62E−13	MAPK9	23	19	0.001634
PRKX	37	36	8.23E−13	ILK	11	11	0.001858
PRKY	39	37	2.82E−12	CAMK2D	51	34	0.002322
CELSR2	41	38	7.28E−12	FRZB	32	24	0.002315
WNT5A	38	36	8.82E−12	PRKCA	35	25	0.005208
TBL1XR1	35	33	2.49E−10	PPAP2B	37	26	0.00568
DVL3	27	27	8.30E−10	CCND1	39	27	0.00611
SLC9A3R1	30	29	1.69E−09	FZD8	27	20	0.010784
TBL1X	42	35	1.10E−07	SFRP4	52	33	0.011249
WNT2B	21	21	3.05E−07	PRKCB	35	24	0.016357
SOSTDC1	46	36	1.48E−06	CCND3	19	15	0.018143
SENP2	26	24	1.69E−06	MMP7	43	28	0.018448
CSNK2A1	20	19	1.98E−05	TGFB1I1	23	17	0.027185
RYK	20	19	1.98E−05	PPP3CA	18	14	0.031241
FZD6	16	16	2.66E−05	SKP1	33	22	0.042493
MYC	25	21	0.000348	DIXDC1	22	16	0.044592
SMAD3	29	23	0.000702				
FOSL1	44	31	0.001016				
KREMEN1	24	19	0.003803				
HHEX	26	20	0.00483				
TP53	9	9	0.007524				
WNT11	9	9	0.007524				
PLCB4	34	24	0.008865				
SMAD2	12	11	0.009335				
TLE1	17	14	0.013585				
DVL2	8	8	0.015954				
RNF138	11	10	0.018424				
VANGL1	11	10	0.018424				
BARX1	21	16	0.02324				
LEF1	16	13	0.024639				
LDB1	10	9	0.035772				

a : number of samples with high expression.

b : number of Group 1 samples with high expression.

c : number of samples with low expression.

d : number of Group 1 samples with low expression.

**Figure 3:**
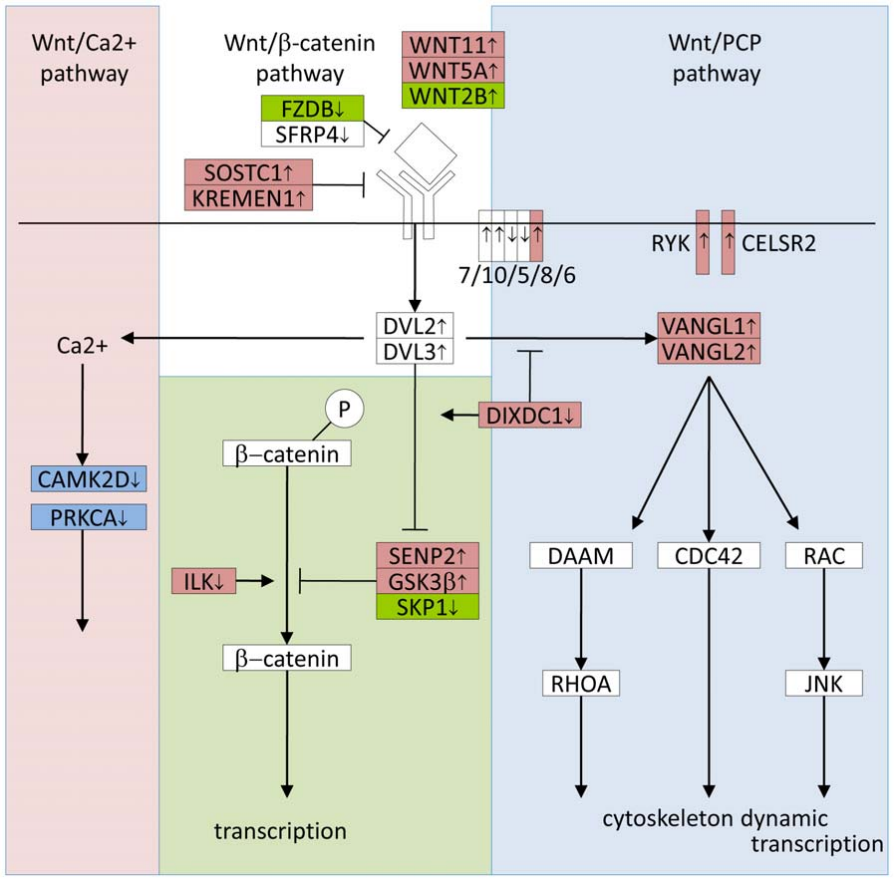
Mapping gene expression changes on the Wnt pathway revealed strong upregulation of PCP signaling and downregulation of canonical signaling. The three branches of Wnt signaling are shown –calcium (pink background), beta-catenin (green background), and PCP (blue background). Up/down arrows indicate overexpression and downregulation, respectively, in Group 1 samples. Genes whose expression change is consistent with canonical pathway inhibition and PCP pathway activation are colored red. Those genes promoting canonical signaling and inhibiting the PCP branch are colored green. Genes with no significant change in expression, or whose expression change has no selectivity between the canonical and non-canonical branches are colored white. The down-regulation of *CAMK2D* and *PRKCA* (dark blue) acts to inhibit Wnt Ca2+ signaling.

More detailed analysis of these genes revealed that the direction of altered expression seemed to indicate an up-regulation of the non-canonical Wnt/PCP pathway and down-regulation of the canonical Wnt/β-catenin signaling branch in Group 1 samples (**Figure 3** and **Table 2**). Several of these findings are summarized here:

- Four genes (*RYK*, *CELSR2*, *VANGL1*, *VANGL2*) described as upstream, positive regulators of the Wnt/PCP pathway [13] were all enriched by CAFET in Group 1 samples.

- Two Wnt ligands, *WNT5A* and *WNT11* [14], were also found overexpressed in Group 1 samples. Both of these Wnt ligands are known to enhance the non-canonical branch of the Wnt pathway while inhibiting the canonical signaling cascade. Of the 38 samples overexpressing *WNT5A*, 37 were found in Group 1. Similarly all nine samples overexpressing *WNT11* were Group 1 samples.

- The Frizzled receptor *FZD6* was exclusively overexpressed among Group 1 samples. This gene was reported to repress canonical Wnt signaling [14], [15] and to activate the non-canonical Wnt pathway [14].

- Samples overexpressing two *LRP6* inhibitors, *SOSTC1* and *KREMEN1*, were enriched among Group 1 samples. The *LRP6* co-receptor is required for canonical Wnt signaling, but not non-canonical signaling [16], [17].

- *DIXDC1* was down-regulated in 21 samples, 16 of which were in Group 1. This gene functions as a switch by enhancing canonical Wnt signaling while inhibiting non-canonical Wnt signaling [18], [19].

- Upregulation of *SENP2*, accompanied by reduced expression of *ILK* in Group 1, indicated a potential increase in β-catenin degradation and inhibition of the canonical signaling cascade [18], [19].

Among all the changes in the Wnt pathway, there were only three changes that specifically indicated a potential activation in canonical signaling: increased expression of *WNT2B*, and the down-regulation of *FRZB* and *SKP1* [20], [21]. In contrast, 12 of the observed changes were consistent with the activation of the non-canonical Wnt pathway or inhibition of the canonical signaling branch. Interestingly, we also saw a decrease expression of *CAMK2D* and *PRKCA*, two components mediating the Wnt/Ca2+ pathway [22].

**Figure 4:**
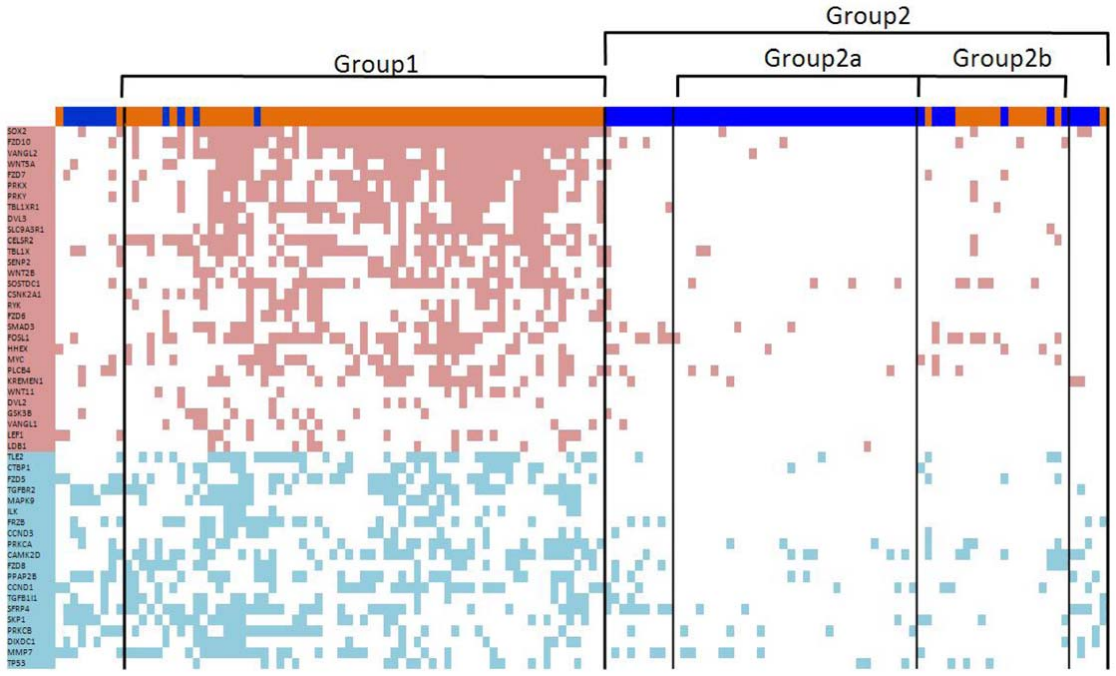
Differential expression pattern of Wnt signature genes in lung cancer samples. Each column represents one of the 138 lung cancer samples as ordered in Fig 1. SCC samples are colored brown and AC samples are colored blue. Each row represents a Wnt pathway gene in Table 2, ranked according to p-value. Red and blue cells indicate overexpression and down-regulation, respectively, of individual genes in specific samples.

**Figure 5:**
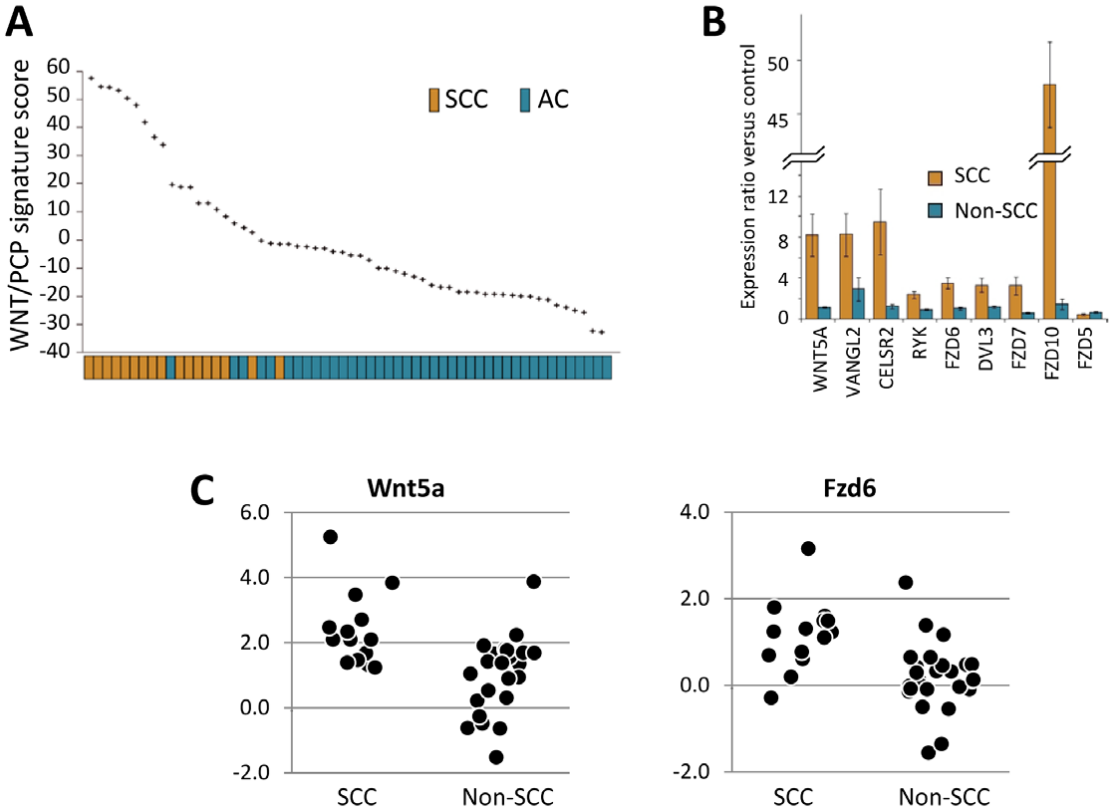
Confirmation of Wnt/PCP signature in SCC of lung. A) Activation of Wnt/PCP signaling in SCC of lung was confirmed in a second independent expression data set. Samples from an additional lung cancer data set [23] were also evaluated for Wnt/PCP signaling using the same scoring system applied to the initial data set. Results showed a strong enrichment of SCC among high-scoring samples. B) Quantitative PCR was also done on three Wnt pathway genes (*FZD6*, *DVL3*, *WNT5A*) in 40 commercially-obtained lung cancer samples. The expression of *WNT5A* and *FZD6*were significantly higher in SCC samples. C) RT-PCR confirmed overexpression of Wnt pathway components in SCC of lung. The expression of nine genes in the Wnt pathway was measured in 12 SCC and 12 non-SCC lung samples. All expression measurements were relative to matched normal lung samples. These data showed consistent upregulation in SCC relative to non-SCC samples. Actin was used as a control for normalization.

These results support the model of increased Wnt/PCP signaling and decreased canonical Wnt signaling in Group 1 SCC samples **(** **Figure 4**). To test the generality of these findings, we examined the role of the Wnt pathway in multiple independent data sets. First, we tested whether the Wnt/PCP signature could be validated in a second, independent gene expression data set [23] and scored the 58 NSCLC samples (40 ACC and 18 SCC) according to the differential expression of genes listed in **Table 2**. Samples with high Wnt/PCP scores were strongly enriched for SCC subtype (**Figure 5A**), confirming a general association between SCC and Wnt/PCP signaling in primary lung tumor sample datasets.

Next, we performed quantitative PCR on three Wnt pathway genes (*FZD6*, *DVL3*, *WNT5A*) in a commercial panel of 40 lung cancer samples (Origene; Rockville, MD). Although the overexpression of *DVL3* did not achieve statistical significance (p = 0.057), *FZD6* and *WNT5A* were both significantly overexpressed in SCC samples relative to the non-SCC samples (p = 0.00056 and p = 0.0011, respectively) (**Figure 5B**).

In a third validation set, we examined a set of freshly obtained primary lung SCC and AC samples (Asterand; Detroit, MI). We probed the expression of nine representative genes of the Wnt/PCP signature (*WNT5A*, *VANGL2*, *CELSR2*, *RYK*, *DVL3*, *FZD5*, *FZD6*, *FZD7* and *FZD10*) using real time RT-PCR (**Figure 5C**). With the exception of *FZD5*, we observed significantly increased expression of the Wnt/PCP components in SCC tumors relative to their matched controls, and also relative to the AC samples.

To determine if the observed WNT/PCP pathway enrichment would translate from *in vivo* primary samples to *in vitro* SCC cell line models, we examined expression of eight differentially expressed genes in four SCC lung cancer cell lines: HARA-1, LK2, NCI-H520 and LUDLU-1. Expression of five of the eight genes (*WNT5A*, *RYK*, *DVL3*, *FZD6*, and *FZD10*) was significantly up-regulated in the four SCC cell line samples relative to the eight non-SCC NSCLC controls (**Figure S1**). These cell lines may offer convenient tools to further investigate the role of the Wnt/PCP signature in SCC, as well as provide models for validation of select Wnt/PCP genes as potential therapeutic targets for SCC progression and metastasis.

## Discussion

Pathway analysis algorithms are aimed at calculating the statistical significance of gene expression changes in order to identify known biological pathways most affected by the observed changes. FGA implicitly assumes that a majority of genes in a pathway need to be overexpressed to activate the pathway. However, dysregulation of one or two genes is often enough to significantly alter cell signaling, and we developed the CAFET algorithm based on this underlying assumption.

By comparison to more well-known FGA enrichment methods, the CAFET approach offers complementary strengths and weaknesses. The statistical power of Fisher’s exact test is highly dependent on the total number of observations being compared. Since FGA tests enrichment along a gene axis, it is most appropriate when the number of genes in a pathway is large but can tolerate a relatively small number of samples. In contrast, CAFET performs enrichment along the sample axis, so statistical power is most dependent on the number of samples being studied. CAFET therefore would not be appropriate when the samples sizes are small. However, when samples sizes are large as in the lung cancer data set examined here, CAFET can accurately interrogate gene sets with relatively few genes that are typically characteristic of specific molecular pathways. Based on these characteristics, it is not surprising that CAFET identified the role of the Wnt pathway in our study while FGA did not. Like other variants of gene set enrichment analysis [24], [25], we believe the CAFET approach will be broadly applicable to pathway enrichment analysis in other large data sets as well.

Many previous studies have explored genomic differences in lung cancer subclasses based on histological distinctions, including differences between AC and SCC (for example [26], [27]). However, in this study, we instead chose to use the results of global, hierarchical clustering of gene expression data to define the comparison groups, effectively stratifying samples based on a molecular rather than histological profile. This approach split the SCC samples into two subclasses, one of which showed greater molecular similarity to AC.

The division of samples based on global hierarchical clustering led to the initial identification by CAFET of the Wnt pathway’s importance in lung cancer. Although CAFET also detected the Wnt pathway as being statistically enriched when simply comparing SCC to AC (data not shown), the subset of SCC in Group 2b was clearly more similar to the AC samples in Group 2a than the remaining SCC samples in Group 1.

Previous reports using gene expression profiling have also noted genomic similarities between AC and certain subsets of SCC [28], [29]. Interestingly, Wilkerson et al. previously characterized a “secretory” subclass of SCC which overexpressed thyroid transcription factor 1 (*NKX2-1/TTF1*), the corresponding protein of which is also highly expressed in AC [30]. In the current study, *NKX2-1/TTF1* also showed significantly higher expression in Group 2 relative to Group 1 samples (p = 1.55E−31), as well as significantly higher expression in Group 2b relative to Group 1 (p = 1.12E−10). These results reinforce the value of studying differences between groups based on genomic profiling rather than histological classification.

Having used the CAFET method to identify a strong enrichment of the Wnt pathway components in NSCLC, we then pursued more detailed characterization. Specifically, we observed a selective up-regulation of the Wnt/PCP pathway, accompanied by potential silencing of the canonical Wnt signaling branch in the SCC subtype. Although the CAFET approach is only applicable in large data sets with many cancer samples, these results demonstrate that CAFET is a powerful and complementary tool for pathway analysis.

The Wnt pathway is highly evolutionarily conserved. Its signaling is initiated by the binding of an extracellular Wnt ligand to a Frizzled-family receptor at the cell surface. Dependent on the specific combination of Wnt/Frizzled isoforms and the presence of specific downstream components, this binding event can signal through three different intracellular branches: the canonical Wnt pathway which terminates in β-catenin-mediated transcription, the Wnt calcium pathway which results in calcium-dependent signaling, and Wnt planar-cell-polarity (PCP) pathway which modulates cytoskeletal dynamics. The latter two are also referred to as non-canonical Wnt pathways [31].

The function of Wnt signaling in healthy adult lung is unclear, however it is hypothesized to play a role in the maintenance of the stem cell niche in the proximal and distal airways [32], [33]. In addition, several groups have linked hyperactivation of Wnt signaling to oncogenesis and metastasis, through dysregulation of such cell processes as cell proliferation, self-renewal capacity, differentiation and cell movement [8], [9].

The canonical branch of the Wnt pathway is by far the most thoroughly studied [8], and has been demonstrated to play a critical role in a wide range of cancers. In colorectal cancer, mutations of β-catenin, APC, and Axin increase the stability of β-catenin, leading to the overexpression of downstream targets and promoting cell proliferation and regulate cell differentiation [9]. Dysregulation of canonical Wnt signaling has also been demonstrated in lung cancer, mediated through epigenetic silencing of negative regulators such as *SFRP1* and *WIF-1* [34], [35] or rare mutations in *APC* and β-catenin [36], [37]. In addition, exposure to cigarette smoke activates the canonical Wnt pathway in human bronchial epithelial cells and induces a tumor-like phenotype [38]. Hyperactivation of the canonical Wnt pathway was also observed in metastatic AC subpopulations and demonstrated to be important for AC metastasis to brain and bone [39].

The non-canonical Wnt pathways, and in particular the Wnt/PCP branch, have been much less studied. Signal transduction from the Wnt/Frizzled/Dsh complex proceeds through unique protein components, including *VANGL*, *PRICKLE*, *CELSR* [40]. This signaling activates the Rho GTPases Rac, Cdc42, and RhoA, which in turn modulate cytoskeleton structure and gene transcription. The Wnt/PCP pathway regulates planar cell polarity as well as the coordinated cell movement in embryos during gastrulation [41]. The Wnt/Frizzled/Dsh complex also stimulates the Wnt calcium pathway by increasing intracellular calcium and activating two calcium dependent kinases, calmodulin-dependent protein kinase II (*CAMKII*) and protein kinase C (*PKC*) [42].

To our knowledge, this is the first observation of the selective enhancement of the Wnt/PCP pathway and inhibition of the canonical Wnt pathway in lung SCC. Although the direct evidence of Wnt/PCP pathway in cancer development is sparse, its potential involvement in tumor progression, angiogenesis, invasion and metastasis has been the focus of recent research. Several groups have identified *WNT5A* as a key regulator of metastasis in melanoma, breast and gastric cancers. In addition both *FZD7* and *FZD10* have been shown to regulate migration and metastasis of gastric, colorectal and synovial carcinomas via the non-canonical Wnt signaling cascade [42], [43]. Furthermore, inhibition of *VANGL1*, an essential component of Wnt/PCP pathway that is overexpressed in SCC, was found to inhibit the size and metastatic potential of gastric tumors in mice [44]. Due to its role in regulating cell adhesion and cell migration in response to microenvironmental cues, it is not surprising the Wnt/PCP pathway is emerging as a central player in tumor invasion and metastasis [42], [43].

Interestingly, the SCC samples in Group 2b do not share the Wnt/PCP pathway signature (**Figure 4**), and every validation data set examined also contained a small number of SCC samples without activation of Wnt/PCP signaling. We are unable to find any secondary correlates that associate with this SCC subclass. The clinical relevance of this subset of SCC samples that lack the Wnt/PCP pathway signature is still an open question, but these findings suggest a potential avenue of study for patient stratification.

Until recently, SCC and AC were treated with a very similar clinical approach [45]. In this study, we present a clear molecular pathway that is highly associated with these two subtypes of. Our analysis indicates that the Wnt/PCP expression is significantly different between a subgroup of SCC and AC. We believe that these results provide important insights on the mechanisms of SCC formation. Moreover, we suggest that targeting individual components of this pathway may also be of therapeutic interest.

## Supporting Information

Table S1 Genes overexpressed in Group 1 samples compared to Group 2 samples.
(XLSX)

Table S2 Genes overexpressed in Group 2 samples compared to Group 1 samples.
(XLSX)

Table S3 Functional groups enriched by FGA analysis among genes overexpressed in Group 1 samples.
(XLSX)

Table S4 Functional groups enriched by FGA analysis among genes overexpressed in Group 2 samples.
(XLSX)

Table S5 Functional groups enriched by CAFET analysis among genes overexpressed in Group 1 samples.
(XLSX)

Table S6 Functional groups enriched by CAFET analysis among genes overexpressed in Group 2 samples.
(XLSX)

Table S7 Functional groups related to the Wnt pathway.
(XLSX)

Table S8 Genes related to the Wnt pathway.
(XLSX)

Figure S1 Expression of Wnt pathway genes in SCC cell lines relative to non-SCC controls. Five of the eight genes examined had significantly higher expression (and lower delta(Ct) values) in SCC samples (p<0.01).
(TIF)
