# Supplementary figures and images for: CAFET Algorithm Reveals Wnt/PCP Signature in Lung Squamous Cell Carcinoma
Source: PLoS One. 2011 Oct 10;6(10):e25807. doi: 10.1371/journal.pone.0025807 (PMC3189939; doi:10.1371/journal.pone.0025807)

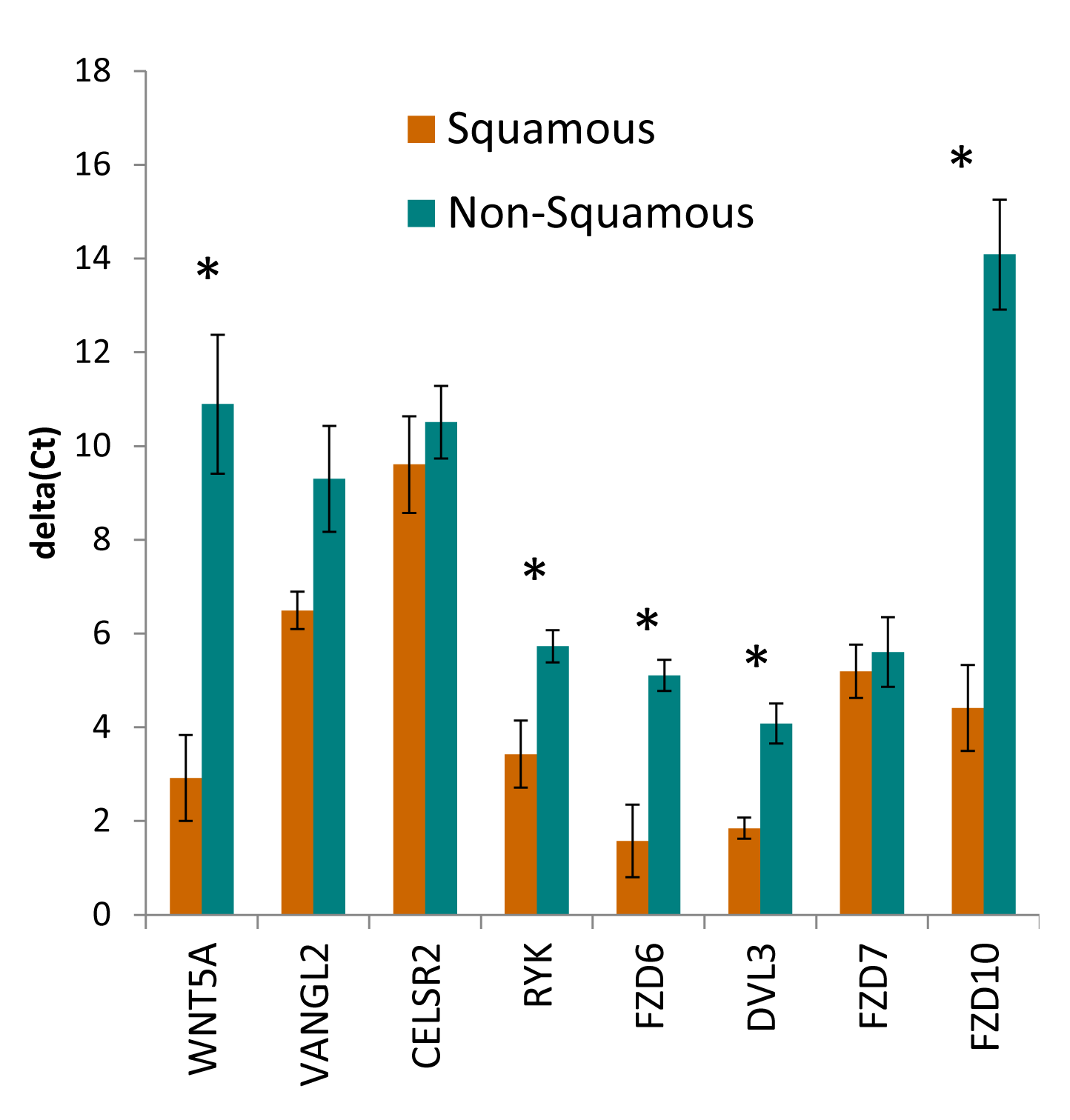

Supplement: Figure S1 — Expression of Wnt pathway genes in SCC cell lines relative to non-SCC controls. Five of the eight genes examined had significantly higher expression (and lower delta(Ct) values) in SCC samples (p<0.01). (TIF) [file pone.0025807.s009.tif]
